# Growth-Associated SNPs of the *NCAPG*-*LCORL* Locus and Divergent Functions of *NCAPG* and *LCORL* in Chicken SMSCs

**DOI:** 10.3390/ani16142244

**Published:** 2026-07-20

**Authors:** Tian Xia, Yuzhu Cao, Qihui Jia, Hongbo Guan, Chenglin Ma, Yulong Guo, Weihua Tian, Xiaojun Liu, Xiangtao Kang, Hong Li

**Affiliations:** 1 College of Animal Science and Technology, Henan Agricultural University, Zhengzhou 450046, China; 2 Henan Key Laboratory for Innovation and Utilization of Chicken Germplasm Resources, Zhengzhou 450046, China; 3 International Joint Research Laboratory for Poultry Breeding of Henan, Zhengzhou 450046, China

**Keywords:** *NCAPG*-*LCORL*, 8-week body weight, SNP, skeletal muscle satellite cells, chicken

## Abstract

Body weight (BW) serves as a core indicator for evaluating chicken growth performance and implementing early individual selective breeding. Our previous studies have verified that 8-week body weight (BW8) exhibits a significantly positive correlation with body weights at 10 and 12 weeks of age in chickens, implying that early body weight can be used to predict late growth phenotypes; nevertheless, the underlying genetic regulatory mechanisms remain elusive. Multiple investigations on growth traits of livestock have confirmed that the *NCAPG*-*LCORL* locus acted as a critical candidate interval associated with growth-related traits. In the present study, an F_2_ segregating population derived from the cross of Gushi chickens and Anka broilers was used as experimental material. Single-nucleotide polymorphism (SNP) screening within the *NCAPG*-*LCORL* locus was performed based on GBS data, followed by the LD analysis. A total of eight SNPs were identified to be significantly associated with economic growth traits in chickens, including four tag SNPs and four non-tag SNPs. In vitro functional validation using chicken skeletal muscle satellite cells (SMSCs) revealed functional divergence between *NCAPG* and *LCORL* during myocyte development: *NCAPG* facilitated SMSCs proliferation while repressing SMSCs differentiation, whereas *LCORL* exerted positive regulatory effects on both proliferation and differentiation of SMSCs. Collectively, *NCAPG* modulates whole-body chicken growth by remodeling the dynamic balance between proliferation and differentiation of skeletal muscle satellite cells. In contrast, *LCORL* simultaneously promotes myocyte proliferation and differentiation to accelerate skeletal muscle development, thereby enhancing chicken growth performance. This study elucidates the regulatory roles of the *NCAPG*-*LCORL* locus in chicken growth at both the molecular marker and cellular functional levels, laying a theoretical foundation for marker-assisted selection of growth traits in chicken breeding.

## Simple Summary

Body weight is a critical indicator to evaluate the growth performance of chickens at all production stages and is a core parameter for early breeding selection. Given that the *NCAPG*-*LCORL* locus has been widely reported to regulate animal body weight and skeletal muscle development, this gene region was selected as the research candidate. Based on genotyping-by-sequencing (GBS) data from an F_2_ resource population, we screened SNPs within the *NCAPG*-*LCORL* locus. After linkage disequilibrium (LD) analysis, association analyses were performed between tag SNPs/non-tag SNPs and 12 economic traits. Furthermore, we explored the regulatory effects of *NCAPG* and *LCORL* on the proliferation and differentiation of chicken skeletal muscle satellite cells (SMSCs) at the cellular level. The elite chickens carrying favorable SNPs identified in this study can be selected in breeding programs to improve economic benefits for poultry farmers.

## 1. Introduction

In China, chicken consumption is second only to pork, and market demand continues to rise [1]. Compared with fast-growing broiler chickens, indigenous chicken breeds are more favored by consumers due to their succulent meat, rich nutrition, and distinctive flavor [2]. Early body weight acts as a crucial indicator of chicken growth and development and serves as an important basis for the early selection of individuals for breeding purposes [3,4]. Owing to the negative genetic correlation between BW and egg production [5,6], in the breeding of maternal lines, the appropriate weight index for chickens can not only maintain favorable body condition and ensure the persistence of egg production performance during the initial laying period, but also exert a genetic impact on the early growth rate of their hybrid offspring.

Therefore, breeders have explored the correlation between early body weight and various traits in many ways to provide a scientific basis for early selection breeding. For example, studies have shown that reduced chicken BW will lead to a significant reduction in egg weight [7], and breeding for increased BW increased egg weight by 11% over nearly 30 generations of turkeys [8]. In addition, growth traits are important indicators in poultry production, and lower BW means higher production costs for broilers [9]. Local chicken breeds exhibit a relatively small body weight, and the genetic variation underlying this weight disparity remains largely unexplored. Consequently, it poses challenges for Chinese indigenous chicken breeds to satisfy market demands.

Growth traits are complex traits controlled by micro-efficient polygenes with moderate heritability and are co-regulated by genetic, nutritional and environmental factors [10]. Therefore, identification of the key genes that affecting growth traits, elucidation of their mechanisms, and development of related molecular markers have been the focus of poultry genetic breeding [11,12]. Accumulating evidence on livestock and poultry growth traits has confirmed that the *NCAPG*-*LCORL* locus acts as a key candidate genomic locus regulating animal growth, body conformation, and carcass traits, exhibiting evolutionary conservation and pleiotropic effects across diverse livestock and poultry species. In ruminant research, Lindholm-Perry et al. conducted SNP genotyping covering the bovine *NCAPG*, *LCORL* and *LAP3* genes and their flanking regions and identified multiple SNPs within the *NCAPG*-*LCORL* locus with strong genetic associations with average daily gain in cattle [13]. Subsequent genome-wide association studies (GWASs) in ewes by Posbergh et al. further validated that this locus exerts major effects on mature body size traits in sheep [14]. Studies focusing on Chinese indigenous beef cattle also demonstrated that *NCAPG* and *LCORL* exert significant genetic effects on bone mass, carcass performance and somatic growth characteristics in Chinese Simmental beef cattle [15]. In addition, the conserved regulatory functions of the *NCAPG*-*LCORL* locus have been widely verified in poultry. Comparative genomic analyses across multiple chicken breeds revealed that the *NCAPG*-*LCORL* genes undergo strong artificial selection during germplasm improvement and evolutionary breeding for body weight in Chinese indigenous chicken breeds [16]. Furthermore, multiple chicken GWASs have confirmed that *NCAPG* and *LCORL* are tightly linked to core growth phenotypes, including slaughter performance, body weight and body morphology, making them core functional genes controlling poultry growth and development. Collectively, the *NCAPG*-*LCORL* locus broadly mediates a wide spectrum of growth-related phenotypes in cattle, sheep and chickens, such as average daily gain, bone mass, adult body size, body weight and carcass traits [17,18]. As an evolutionarily conserved core locus governing animal growth, it serves as a vital target to dissect the molecular genetic basis of growth traits in livestock and poultry.

Gushi chicken is a famous local chicken breed in China used for both meat and eggs. Because of its excellent meat quality and unique flavor, it is often used as a material for high-quality broiler breeding [19]. However, the weight of the culled chickens is too small to meet the market demand. Therefore, it is particularly urgent to improve the growth performance of Gushi chickens and improve the weight of culled chickens. Through the implementation of early selection, the problem of high production costs caused by large seed retention can be reduced, and the production efficiency can be improved [20,21]. Accordingly, this study focuses on the *NCAPG*-*LCORL* locus to screen key functional variants regulating chicken growth and development and to investigate the regulatory effects of this evolutionarily conserved locus on the proliferation and differentiation of chicken skeletal muscle satellite cells, aiming to provide theoretical support and candidate molecular targets for marker-assisted selection in livestock and poultry breeding.

## 2. Materials and Methods

### 2.1. Animal Ethics

All animals used in this study complied with animal welfare requirements, and the experimental procedures were conducted under the approval of the Institutional Animal Care and Use Committee of Henan Agricultural University (protocol code No. 11-0085, approval date: 24 May 2022).

### 2.2. Samples Collection

Breast muscle and leg muscle samples from Gushi chickens at 2, 4, 6, and 8 weeks of age were harvested and rapidly frozen in liquid nitrogen, subsequently stored at −80 °C, with six birds sampled at each time interval. Additionally, heart, liver, spleen, lung, and duodenum samples from Gushi chickens at 8 weeks of age were collected, flash frozen in liquid nitrogen, and thereafter stored at −80 °C.

### 2.3. Resource Populations and Traits for Association Analysis

The F_2_ resource group of Gushi × Anka chicken was constructed by Henan Innovative Engineering Research Center of Poultry Germplasm Resource, and this population was designed according to the F_2_ distant half-sib, with 7 crossover families; the specific construction scheme of this resource group was described by Han et al. [22]. A total of 860 chickens were freely fed and given water in the same environment. During this period, weight was weighed every 2 weeks, body size index was measured every 4 weeks, and phenotypic index was measured when the neck was dislocated at 84 days of age. All the detailed measuring methods were described previously [23]. GBS data of the 860 individuals were obtained from our previous description [24].

### 2.4. Overexpression Vector Construction for Candidate Genes

The coding region sequence of the chicken *NCAPG* gene (XM_025150356.3) and *LCORL* gene (NM_001031160.3) were obtained from the NCBI database, and the homology arm primers were designed using an online website (https://crm.vazyme.com/cetool/singlefragment.html, accessed on 20 May 2023). The coding region of *NCAPG* was amplified by PCR and cloned into the pcDNA3.1(+) (Invitrogen, Carlsbad, CA, USA) vector linearized by *NheI* (NEB, Ipswich, MA, USA) and *BamHI* (NEB). The *NCAPG* overexpression vector was constructed and designated as *pcDNA3.1*-*NCAPG*. Meanwhile, the coding region of *LCORL* was amplified by PCR and cloned into the pcDNA3.1(+) vector linearized by *HindIII* (NEB) and *EcoRI* (NEB). The *LCORL* overexpression vector was constructed and named pcDNA3.1-LCORL. The primer information is shown in Supplementary Table S1.

### 2.5. Chicken Skeletal Muscle Satellite Cells (SMSCs) Isolation, Culture and Treatment

Based on the method reported by Baquero-Perez et al. [25] and Han et al. [26], SMSCs were isolated from breast muscles on the 14th day of AA chicken embryos. Briefly, chicken SMSCs were obtained using a mixed enzyme digestion method and differential attachment method and were cultured in a solution containing 10% fetal bovine serum (BioInd, Kibbutz Beit Haemek, Israel) and 1% penicillin–streptomycin (Solarbio, Beijing, China) in DMEM/F12 medium (BioInd, Kibbutz Beit Haemek, Israel). Cells were cultured at 37 °C with 5% CO_2_.

### 2.6. RNA Extraction, cDNA Synthesis and Quantitative Real-Time PCR (qRT–PCR)

Total RNA was isolated from tissue and skeletal muscle satellite cells according to the instructions of Trizol Reagent (Vazyme Biotech, Nanjing, China). The RNA concentration and integrity was evaluated using NanoDrop 2000 spectrophotometer (Thermo Scientific, Wilmington, DE, USA) and 1% agarose gel electrophoresis, respectively. The cDNA was synthesized according to the instructions of the HiScript reverse transcription kit (Vazyme Biotech, Nanjing, China).

qRT-PCR was performed on a LightCycler^®^96 instrument (Roche, Basel, Switzerland) using the SYBR Green method according to the instructions of ChamQ Universal SYBR qPCR Master Mix (Vazyme Biotech, Nanjing, China) with glyceraldehyde-3-phosphate dehydrogenase (*GAPDH*) as the reference gene. For qRT-PCR amplification of the collected tissues, each sample was analyzed with six biological replicates and three technical replicates. Statistical evaluation did not consider intra-sample variation.

Each reaction system included 2 × ChamQ Universal SYBR qPCR Master Mix 5 μL, upstream and downstream primers 0.5 μL (10 μM) each, cDNA 1 μL, and RNase-free water (Solarbio, Beijing, China) 3 μL. The qRT-PCR reaction program was set as follows: pre-denaturation at 95 °C for 5 min, denaturation at 95 °C for 30 s, annealing at the optimum temperature of 60 °C for 30 s, extension at 72 °C for 30 s, 40 cycles, and final extension of 72 °C for 10 min. Gene primers were designed using the NCBI Primer-BLAST tool (https://www.ncbi.nlm.nih.gov/tools/primer-blast/; accessed on 10 June 2023), and the primer sequences were synthesized by Tsingke Biotech Co., Ltd. (Beijing, China). Primer information is shown in Supplementary Table S2.

### 2.7. Cell Proliferation Assays

At 12, 24, 36, and 48 h after plasmid transfection, cell viability was assessed using the Cell Counting Kit-8 (CCK-8) (Vazyme Biotech, Nanjing, China) following the manufacturer’s instructions. Subsequently, the absorbance was measured at a wavelength of 450 nm using a Multimode Reader (BioTek, Winooski, VT, USA) after incubation for 2 h.

After transfection with SMSCs for 24 h, cell proliferation was assessed using the Cell-Light^TM^ EdU Apollo In Vitro Kit (RiboBio, Guangzhou, China), images were taken with Inverted Fluorescence Microscope (Olympus, Tokyo, Japan), and cell proliferation was analyzed with ImageJ software (version 1.52a).

The SMSCs were collected after transfection for 24 h and fixed in pre-cooled 80% ethanol at −20 °C overnight. The cells were washed with pre-cooled PBS, and the supernatant was discarded after centrifugation. The cell cycle kit (KeyGEN Biotech, Nanjing, China) was determined using BD AccuriC6 flow cytometry (BD Biosciences, Franklin Lakes, NJ, USA). The cell cycle was analyzed by ModFit software (version 5.0).

### 2.8. Immunofluorescence Analysis

The cells were inoculated into 24-well plates. After transfection with the plasmid for 48 h, the cells were fixed with 4% formaldehyde for 30 min. MyHC antibody (murine, 1:500, DSHB) and Cy3 antibody (goat anti-mouse, 1:100, DSHB) were used for immunofluorescence, nuclear staining was used for DAPI, and MyHC immunofluorescence was used for muscle duct formation analysis.

### 2.9. Statistical Analysis

The association analysis between the economic traits of the F_2_ resource population and different genotypes or haplotype combinations of *NCAPG*-*LCORL* were performed using the mixed linear models (least squares analysis) of SPSS 24.0 software (IBM, Chicago, IL, USA). Multiple comparisons were done using Bonferroni’s correction. Model I was used for growth traits. Carcass weight was used as a covariate, and Model II was used for carcass traits based on the effect of carcass weight on carcass traits. The model for association analysis was as follows:

Model I: Y_ijklm_ = µ + G_i_ + S_j_+ H_k_ + f_l_ + e_ijklm_

$$\mathrm{Model}\;\mathrm{II}:\;Y_{\mathrm{ijklm}}=µ+G_{i}+S_{j}+H_{k}+f_{l}+b\;(W_{\mathrm{ijklm}}-\overset{-}{W})+e_{\mathrm{ijklm}}.$$

In the model formula, Y_ijklm_ represents the phenotypic value of individual traits; µ is the overall mean value of traits. G_i_ is the fixed effect of genotype (1–3). S_j_ is a fixed effect of sex (1, 2); H_k_ is the fixed effect of the batch (1, 2); f_l_ denotes random family effect (1–7); b refers to the regression coefficient of carcass weight; e_ijklm_ stands for the random error; W_ijklm_ is individual slaughter weight; $\overset{-}{W}$ is the average slaughter weight.

The 2^−ΔΔCt^ method was used to analyze the relative expression level of genes. Multiple comparisons in SPSS 24.0 were used to examine the significance of gene relative expression levels between different tissues. One-way analysis of variance was used to compare the significance of relative expression levels of genes in the same tissue at different stages. Data visualization was performed using GraphPad Prism 8.0 (San Diego, CA, USA) software. All data are presented as mean ± standard error of the mean (SEM). Statistical significance was defined as follows: *p* < 0.05 (*) indicated a significant difference; *p* < 0.01 (**) indicated extremely significant differences; and *p* ≥ 0.05 indicated no significant difference.

## 3. Results

### 3.1. Identification of SNP Variants in the Chicken NCAPG–LCORL Locus

**Figure 1:**
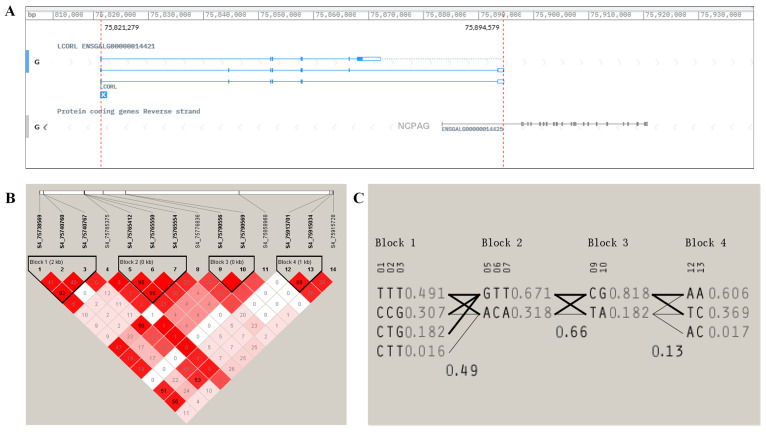
*NCAPG*-*LCORL* locus-related genetic characteristics. (**A**) Genomic location relationship of *NCAPG* and *LCORL* from the Ensembl database. Red dashed lines denote the genomic boundaries of the *LCORL* gene. (**B**) LD analysis of SNPs in *NCAPG–LCORL* locus. The intensity of red color indicates the strength of pairwise LD, where deeper red represents higher r^2^ values (**C**) Haplotype combinations of SNPs on the *NCAPG*-*LCORL* gene locus.

The Ensemble database shows that *NCAPG* (ENSGALG00000014425) and *LCORL* (ENSGALG00000014421) overlap in the transcript of two genes on the genome (Figure 1A). Subsequently, 44 SNP loci on the *NCAPG*-*LCORL* gene were screened from GBS data of F_2_ resource group “Gushi × Anka”, all of which were located on introns, among which only 14 SNPs were significantly associated with BW8 (*p* < 0.05; Supplementary Table S3). LD analysis of these 14 candidate SNPs showed that 10 SNPs were located in 4 block modules. We selected one representative tag SNP from each block: rs75738569, rs75765412, rs75790569 and rs75915034. The SNPs outside the LD blocks were regarded as non-tag SNPs, including rs75765375, rs75776836, rs75858968 and rs75915728. Based on the reference genome *Gallus gallus* 6.0, the nucleotide positions of these SNPs are rs75738569 (Chr4, 75738569), rs75765412 (Chr4, 75765412), rs75790569 (Chr4, 75790569), rs75915034 (Chr4, 75915034), rs75765375 (Chr4, 75765375), rs75776836 (Chr4, 75776836), rs75858968 (Chr4, 75858968), rs75915728 (Chr4, 75915728). These eight SNPs were used to further screen molecular markers related to chicken growth and development (Figure 1B,C).

### 3.2. Association Analysis Between Candidate Tag SNPs of NCAPG–LCORL Gene Locus and Economic Traits in F_2_ Resource Population

**Figure 2:**
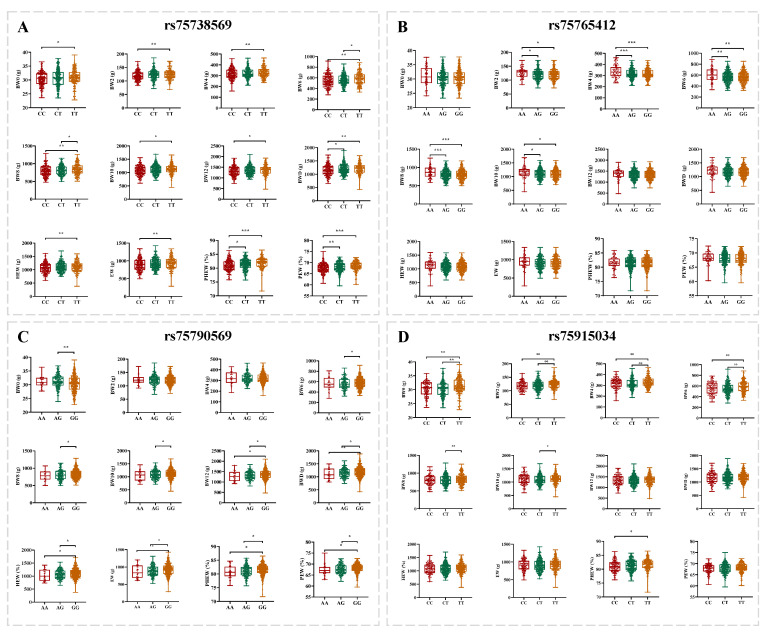
Correlation analysis of 4 tag SNPs and 12 economic traits. (**A**–**D**) Correlation analysis of rs75738569, rs75765412, rs75790569, rs75915034 and 12 economic traits of F_2_ resource population. The 12 economic traits include 0-week body weight (BW0), 2-week body weight (BW2), 4-week body weight (BW4), 6-week body weight (BW6), BW8, BW10, BW12, body weight after depilation (BWD), half-eviscerated weight (HEW), eviscerated weight (EW), percentage of half-eviscerated weight (PHEW), and percentage of eviscerated weight (PEW). Data are presented as mean ±  SEM. * means *p* < 0.05, ** means *p* < 0.01, *** means *p* < 0.001.

The four tag SNPs were associated with 12 economic trait phenotypes of the F_2_ resource population. The results showed that the three genotypes CC (*n* = 218), CT (*n* = 290) and TT (*n* = 211) of the *NCAPG* genetic variant locus (rs75738569) were significantly associated with the 12 phenotypes BW0~BW12, BWD, HEW, EW, PHEW and PEW (*p* < 0.05), and the growth and slaughter traits of TT genotype individuals were significantly higher than those of CC genotype individuals (*p* < 0.05; Figure 2A). The three genotypes AA (*n* = 71), AG (*n* = 323) and GG (*n* = 325) of the *NCAPG* genetic variation locus (rs75765412) were significantly associated with the five phenotypes of BW2~BW10 (*p* < 0.05), and the body weights of AA genotype individuals were significantly higher than those of AG and GG genotype individuals (*p* < 0.05; Figure 2B). The three genotypes AA (*n* = 34), AG (*n* = 194) and GG (*n* = 491) of the *NCAPG* genetic variation locus (rs75790569) were significantly associated with the 10 phenotypes of BW0, BW6~BW12, BWD, HEW, EW, PHEW and PEW (*p* < 0.05); the BW6~BW12 of GG genotype individuals were significantly higher than those of AG genotype individuals (*p* < 0.05); and the body weight after depilation (BWD), half-eviscerated weight (HEW), eviscerated weight (EW) and percentage of half-eviscerated weight (PHEW) of AA genotype individuals were significantly higher than those of AG and GG genotype individuals (*p* < 0.05; Figure 2C). The three genotypes CC (*n* = 126), CT (*n* = 303) and TT (*n* = 290) of the *NCAPG* genetic variation locus (rs75915034) were significantly associated with the seven phenotypes BW0~BW10 and PHEW (*p* < 0.05), and the BW0~BW8 of TT genotype individuals were significantly higher than those of CC and CT genotype individuals (*p* < 0.05; Figure 2D).

### 3.3. Association Analysis Between Candidate Non-Tag SNPs of NCAPG–LCORL Gene Locus and Economic Traits in F_2_ Resource Population

**Figure 3:**
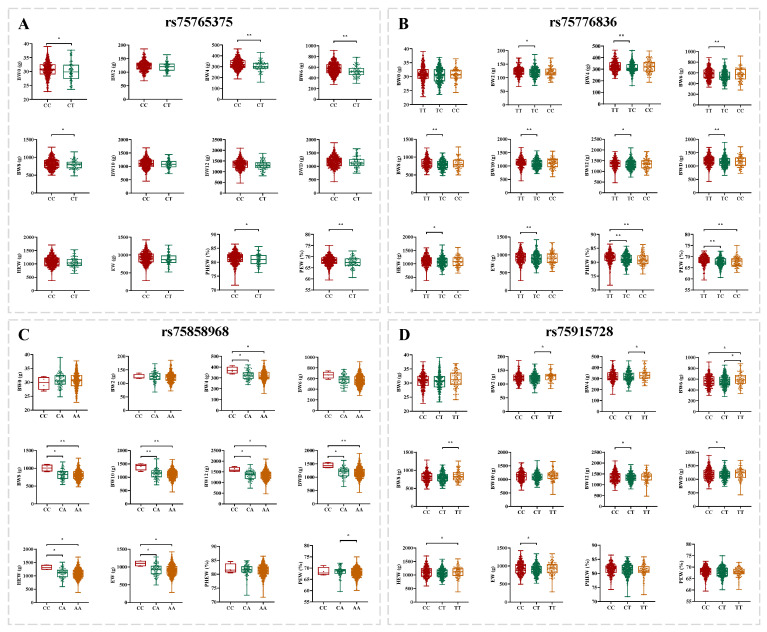
Correlation analysis of 4 non-tag SNPs and 12 economic traits. (**A**–**D**) Correlation analysis of rs75765375, rs75776836, rs75858968, rs75915728 and 12 economic traits of F_2_ resource population. The 12 economic traits include 0-week body weight (BW0), 2-week body weight (BW2), 4-week body weight (BW4), 6-week body weight (BW6), BW8, BW10, BW12, body weight after depilation (BWD), half-eviscerated weight (HEW), eviscerated weight (EW), percentage of half-eviscerated weight (PHEW), and percentage of eviscerated weight (PEW). Data are presented as mean ± SEM. * means *p* < 0.05, ** means *p* < 0.01.

The four non-tag SNPs were significantly associated with 12 economic trait phenotypes in the F_2_ resource population. The results showed that the two genotypes CC (*n* = 628) and CT (*n* = 91) of the *NCAPG* genetic variation locus (rs75765375) were significantly associated with the six phenotypes BW0, BW4~BW10, PHEW and PEW (*p* < 0.05), and the body weights and slaughter traits of CC genotype individuals were significantly higher than those of CT genotype individuals (*p* < 0.05; Figure 3A). The 3 genotypes TT (*n* = 338), TC (*n* = 278) and CC (*n* = 103) of the *NCAPG* genetic variation locus (rs75776836) were significantly associated with the 11 phenotypes of BW2, BW6~BW12, BWD, HEW, EW, PHEW and PEW (*p* < 0.05). The body weights and slaughter traits of TT genotype individuals were significantly higher than those of TC genotype individuals (*p* < 0.05; Figure 3B). The three genotypes CC (*n* = 4), CA (*n* = 80) and AA (*n* = 635) of *NCAPG*-*LCORL* genetic variation locus (rs75858968) were significantly associated with the eight phenotypes of BW4, BW8~BW12, BWD, HEW, EW and PEW (*p* < 0.05), and the body weights and slaughter traits of CC genotype individuals were significantly higher than those of CA and AA genotype individuals (*p* < 0.05; Figure 3C). The three genotypes CC (*n* = 314), CT (*n* = 312) and TT (*n* = 89) of the *NCAPG* genetic variation locus (rs75915728) were significantly associated with the eight phenotypes BW2~BW8, BW12, BWD, HEW and EW (*p* < 0.05), and the body weight of the TT genotype was significantly higher than that of the CT genotype (*p* < 0.05; Figure 3D).

### 3.4. Tissue and Temporal Expression Characteristics of NCAPG and LCORL Genes

**Figure 4:**
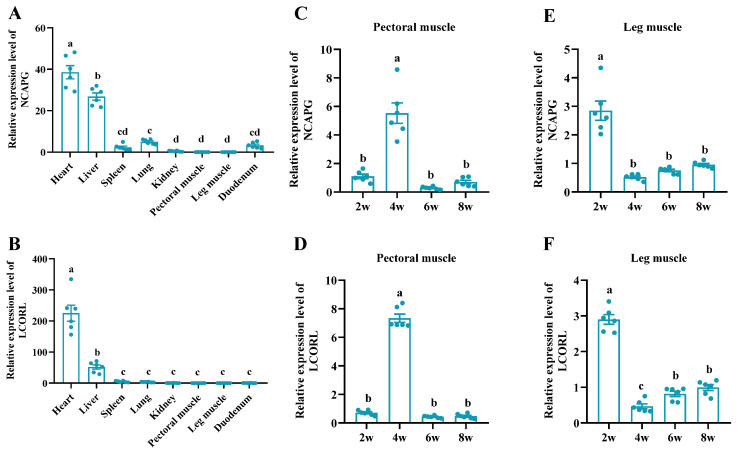
*NCAPG*/*LCORL* gene tissue and spatiotemporal expression profiles. (**A**,**B**) The relative expression of *NCAPG*/*LCORL* in different tissues (*n* = 6). (**C**,**D**) The relative expression of *NCAPG*/*LCORL* in pectoral muscle of chickens at different stages (*n* = 6). (**E**,**F**) The relative expression of *NCAPG*/*LCORL* in leg muscle of chickens at different stages (*n* = 6). Data are presented as mean ± SEM. Different lowercase letters indicate significant difference (*p* < 0.05); the same letters show no significant difference (*p* > 0.05).

To characterize the expression patterns of *NCAPG* and *LCORL*, we quantified their mRNA levels across multiple tissues from 8w Gushi chickens, as well as in pectoral and leg muscles at distinct developmental stages (2, 4, 6, and 8 weeks). Tissue profiling revealed that both *NCAPG* and *LCORL* exhibited markedly higher expression in the heart and liver than in other tissues; *NCAPG* was weakly expressed in the pectoral muscle and leg muscle (Figure 4A,B). In pectoral muscle, the expression of both genes followed an up-then-down pattern with increasing age, peaking at 4 weeks. Their transcript levels at 4 weeks were significantly higher than at all other time points (*p* < 0.05), whereas no significant differences existed among the remaining ages (*p* > 0.05; Figure 4C,D). By contrast, in leg muscle, *NCAPG* and *LCORL* displayed a down-then-up pattern over time, peaking at 2 weeks. Levels at 2 weeks were significantly higher than those at other time points (*p* < 0.05), while no statistical differences were observed among the other sampling weeks (*p* > 0.05; Figure 4E,F).

### 3.5. Effect of NCAPG on SMSCs Proliferation and Differentiation

**Figure 5:**
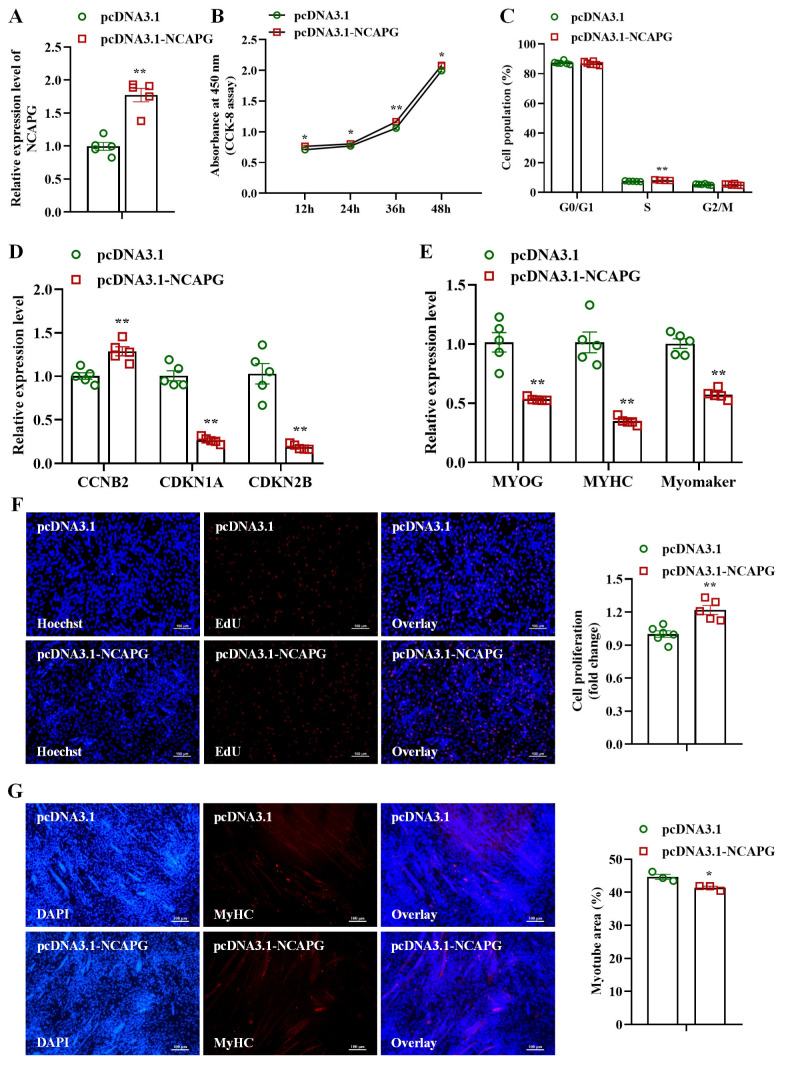
*NCAPG* promotes proliferation and inhibits differentiation of SMSCs. (**A**) The overexpression efficiency of *NCAPG* gene (*n* = 5). (**B**) CCK-8 was used for determining cell viability after SMSCs transfection with pcDNA3.1-NCAPG (*n* = 10). (**C**) The cell cycle analysis of SMSCs overexpressing *NCAPG* was performed by flow cytometry (*n* = 6). (**D**) Effect of overexpression of *NCAPG* on the expression of cell proliferation-related genes (*n* = 5). (**E**) Effect of overexpression of *NCAPG* on the expression of cell differentiation-related genes (*n* = 5). (**F**) EdU staining was performed to detect the proliferation of chicken SMSCs, and the histogram shows the percentage of EdU-positive SMSCs. (**G**) Immunofluorescence staining of MyHC (red) was performed in SMSCs, and the histogram quantifies the MyHC-positive area. Scale bar: 100 μm. Data are presented as mean ± SEM. * means *p* < 0.05, ** means *p* < 0.01.

In order to investigate the role of *NCAPG* in the proliferation and differentiation of chicken SMSCs, *NCAPG* was overexpressed in SMSCs (Figure 5A). CCK-8 assay results demonstrated that *NCAPG* overexpression significantly promoted the proliferation of SMSCs (*p* < 0.05; Figure 5B). Flow cytometry analysis revealed that *NCAPG* overexpression increased the proportion of cells in the S phase (*p* < 0.01; Figure 5C). Furthermore, the expression of the cell proliferation marker gene cyclin B2 (*CCNB2*) was significantly upregulated (*p* < 0.01), whereas the expression of the anti-proliferative marker genes cyclin-dependent kinase inhibitor 1A (*CDKN1A*) and cyclin-dependent kinase inhibitor 2B (*CDKN2B*) were significantly downregulated (*p* < 0.01; Figure 5D). Furthermore, qPCR results revealed that *NCAPG* overexpression markedly suppressed the expression of three myogenic differentiation markers. Relative to the control group, the transcript levels of *MYOG* (myogenin), *MYHC* (myosin heavy chain), and *Myomaker* were reduced 0.53-, 0.34-, and 0.57-fold, respectively, with all differences being statistically significant (*p* < 0.01; Figure 5E). The EdU incorporation assay showed that *NCAPG* overexpression significantly increased the number of EdU-positive cells compared with the control group (*p* < 0.01; Figure 5F). Immunofluorescence staining further revealed that the myotube area was significantly reduced in the *NCAPG* overexpression group compared with the control group (*p* < 0.05; Figure 5G).

### 3.6. Effect of LCORL on SMSC Proliferation and Differentiation

**Figure 6:**
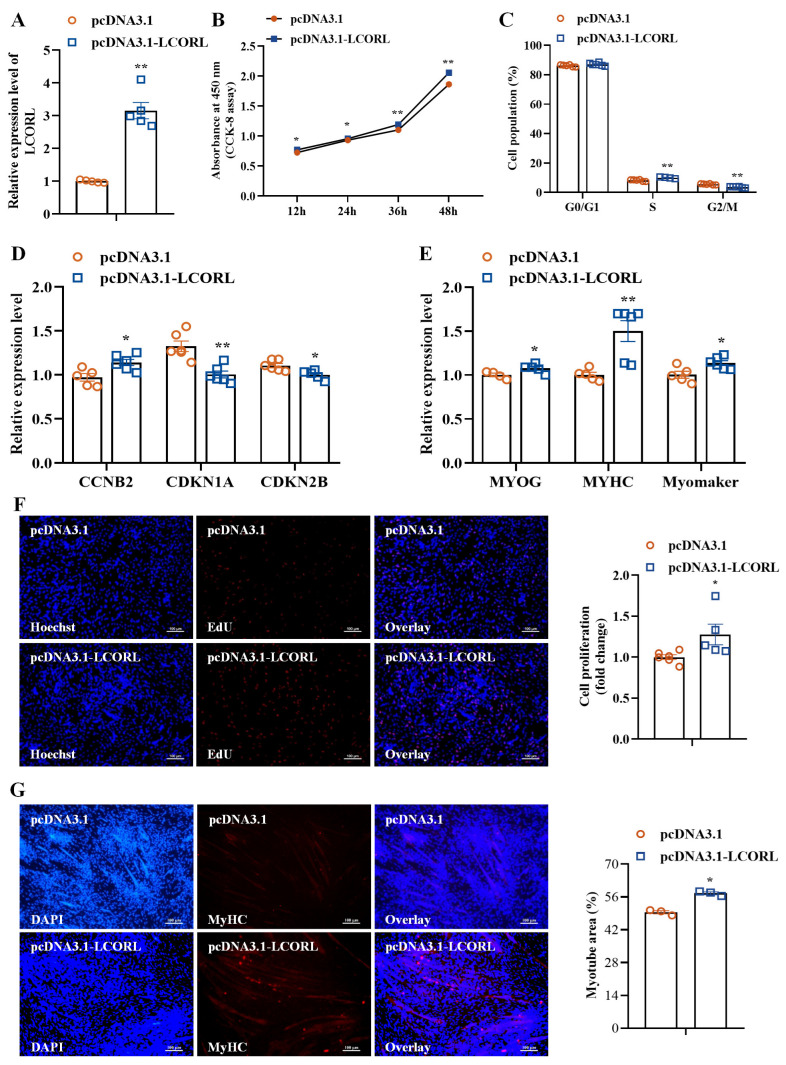
*LCORL* promotes proliferation and differentiation of SMSCs. (**A**) The overexpression efficiency of the *LCORL* gene (*n* = 5). (**B**) CCK-8 was used for determining cell viability after SMSCs transfection with pcDNA3.1-LCORL (*n* = 10). (**C**) The cell cycle analysis of SMSCs overexpressing *LCORL* was performed by flow cytometry (*n* = 6). (**D**) Effect of overexpression of *LCORL* on the expression of cell proliferation-related genes (*n* ≥ 5). (**E**) Effect of overexpression of *LCORL* on the expression of cell differentiation-related genes (*n* ≥ 4). (**F**) EdU staining was performed to detect the proliferation of chicken SMSCs, and the histogram shows the percentage of EdU-positive SMSCs. (**G**) Immunofluorescence staining of MyHC (red) was performed in SMSCs, and the histogram quantifies the MyHC-positive area. Scale bar: 100 μm. Data are presented as mean ± SEM. * means *p* < 0.05, ** means *p* < 0.01.

To investigate the functional role of *LCORL* in the proliferation and differentiation of chicken SMSCs, *LCORL* was successfully overexpressed in SMSCs (*p* < 0.01; Figure 6A). CCK-8 results show that *LCORL* overexpression significantly promoted cell proliferation (*p* < 0.05; Figure 6B). Flow cytometry analysis revealed that *LCORL* overexpression increased the proportion of cells in S phase, accompanied by a significant decrease in the G2/M phase population, indicating an overall acceleration of the cell cycle (Figure 6C). Furthermore, the proliferative marker *CCNB2* was significantly upregulated (*p* < 0.05), while the anti-proliferative markers *CDKN1A* and *CDKN2B* were significantly downregulated (*p* < 0.05; Figure 6D). qRT-PCR analysis further revealed that *LCORL* overexpression markedly increased the mRNA expression of three pivotal myogenic differentiation markers. Compared with the control group, the transcript levels of *MYOG*, *MYHC*, and *Myomaker* exhibited 1.06-, 1.44-, and 1.30-fold increases, respectively, with all differences being statistically significant (*p* < 0.05; Figure 6E). EdU incorporation assay showed that the number of EdU-positive cells was significantly increased in the *LCORL* overexpression group compared with the control group (*p* < 0.05; Figure 6F). Consistently, immunofluorescence staining confirmed that *LCORL* overexpression substantially enlarged the total myotube area (*p* < 0.05; Figure 6G).

## 4. Discussion

In recent years, the *NCAPG*-*LCORL* region has been studied in mammals (humans, cattle, pigs, horses, sheep, etc.) and has been identified as a relevant locus for growth and development traits [13,27,28]. It has been reported that *NCAPG* and *LCORL* genes are located in an important QTL region on GGA4, which may be related to growth traits in the chicken genome [29]. For example, Liu et al. identified a candidate gene *LCORL* in GGA4 that affects carcass weight (CW) and eviscerated weight (EW) through GWAS analysis [30]. In previous research, statistical and correlation analyses of 32 growth traits and 37 carcass traits in the F_2_ resource population showed strong phenotypic correlations between body weight and body size traits at the middle and late growth stages [24]. Meanwhile, study has located the 17 growth and carcass phenotypes, including BW8, on GGA4 and identified *NCAPG* and *LCORL* genes [24]. Subsequently, studies in mice showed that the metabolic results after *LCORL* knockout were consistent with many metabolic parameters reported in human and livestock GWASs [31]. The above-mentioned studies suggest that the *NCAPG*-*LCORL* genes may play an important role in the growth of livestock and poultry, but the function of *NCAPG*-*LCORL* genes in the growth and development of chickens is still unclear.

In livestock and poultry research, many studies have proved that SNP molecular markers have been widely used in breeding [32,33]. The results of this study show that 14 candidate SNPs on the chicken *NCAPG*-*LCORL* gene were significantly correlated with BW8. In addition, although rs75859000 (G > A) is not significantly correlated with BW8, it has been reported to be significantly correlated with other early body weight, body size and carcass traits [34]. The phenotype of animals could be affected by both a single mutation site and a combination of multiple mutation sites, with the latter having a more significant effect [35]. Because single SNP analysis is less useful when studying the impact of genetic variation on phenotype, haplotype analysis can provide richer information [36], but no better haplotype combination was found in this study. A large number of studies have shown that single SNPs in *NCAPG* and *LCORL* genes are significantly correlated with body weight, carcass, meat quality and body size of livestock and poultry [13,37,38,39]. In the present study, eight SNPs identified within the *NCAPG*-*LCORL* gene locus were found to be significantly associated with growth and developmental traits in the F_2_ resource population. These loci are expected to serve as robust and effective molecular markers in local chicken breeding programs, thereby providing an important genetic basis for genetic improvement.

In this study, the chicken *NCAPG* and *LCORL* genes were widely expressed across various tissues, suggesting that they may exert distinct biological functions in different organs. Notably, both genes showed relatively low expression in skeletal muscle, whereas their transcript levels were much higher in the heart and liver. Consistent expression patterns of these two genes were observed in the pectoral and leg muscles of chickens aged 2 to 8 weeks. We speculate that these two genes influence chicken growth-related economic traits through two possible pathways. On the one hand, their high expression in the liver and heart enables them to regulate whole-body metabolism, nutrient transport, and energy supply, thereby indirectly modulating skeletal muscle development and body weight. On the other hand, although their expression in skeletal muscle is low, the detectable transcripts suggest that they participate in the fine-tuning of proliferation and differentiation of skeletal muscle satellite cells during early skeletal muscle development.

We overexpressed *NCAPG* and *LCORL* at the cellular level to study the role of *NCAPG* and *LCORL* in the proliferation and differentiation of chicken SMSCs. The results indicated that *NCAPG* overexpression can promote cell proliferation and inhibit cell differentiation, while *LCORL* overexpression can promote cell proliferation and differentiation. In human studies, *NCAPG* and *LCORL* can promote the proliferation of many cell types, such as glioblastoma (GBM) [40], colorectal cancer cells [41], and other tumor cells [42,43]. Studies have shown that *NCAPG* promotes the proliferation of fetal bovine myoblasts [44] and inhibits the differentiation of fetal bovine myoblasts [45]. Interference with *NCAPG* inhibits proliferation and differentiation of sheep embryonic myoblasts [46]. *NCAPG* reportedly plays a role in sister chromatid segregation [47]. In this study, *NCAPG* and *LCORL* significantly promoted the S-phase number of chicken SMSCs. Therefore, we speculate that chicken *NCAPG* and *LCORL* regulate cell proliferation through DNA replication in the S-phase. It has been reported that *NCAPG* may regulate embryonic muscle development through chromatin accessibility [45,48], while the mechanism by which *LCORL* modulates cell differentiation remains poorly understood. We speculate that chicken *NCAPG* controls cell differentiation via chromatin accessibility, and further investigations are required to elucidate the regulatory mechanism of *LCORL* in this process. Notably, *NCAPG* and *LCORL* exert distinct and synergistic effects on myoblast proliferation and differentiation: *NCAPG* likely acts as a molecular “brake” to suppress premature differentiation and guarantee adequate cell proliferation before myoblast fusion, whereas *LCORL* functions as an “accelerator” to drive terminal myogenic differentiation. Their divergent functions reveal the complex interplay between these two genes during muscle development. Combined with the prediction from the STRING database that there may be an interaction relationship between NCAPG and LCORL proteins, this also provides valuable insights for in-depth research on the regulation of muscle development by the chicken *NCAPG*-*LCORL* gene.

## 5. Conclusions

In this study, we performed SNP screening using GBS data and identified four tag SNPs and four non-tag SNPs in the *NCAPG*-*LCORL* genomic interval significantly associated with growth traits in the Gushi × Anka F_2_ resource population. Functional assays revealed distinct regulatory effects of these two genes on chicken SMSCs: overexpression of *NCAPG* accelerated SMSCs proliferation but suppressed cell differentiation, while overexpression of *LCORL* promoted both proliferation and differentiation of SMSCs.

## Data Availability

The original contributions presented in this study are included in the article/Supplementary Materials. Further inquiries can be directed to the corresponding author.

## Supplementary Materials

The following supporting information can be downloaded at: https://www.mdpi.com/article/10.3390/ani16142244/s1, Table S1: PCR amplification primers; Table S2: Primer sequence used for qRT-PCR; Table S3: Chicken *NCAPG*-*LCORL* gene mutation site information and association results with BW8.
