# Supplementary material for: Growth-Associated SNPs of the NCAPG-LCORL Locus and Divergent Functions of NCAPG and LCORL in Chicken SMSCs
Source: Animals (Basel). 2026 Jul 20;16(14):2244. doi: 10.3390/ani16142244 (PMC13406134; doi:10.3390/ani16142244)
Supplement: Supplementary file 1 [file animals-16-02244-s001.zip › animals-4400632-supplementary.pdf]

Table S1 PCR amplification primers

Gene	Primer Sequence (5'-3')	Length (bp)
NCAPG-CDS	F: gggagacccaagctggctagcATGTGTCCGGCGAAGAAACC<br>R: ccacactggactagtggatccTCATTCATCTGCTTCCGTACGC	3117
LCORL-CDS	F: ctagcgtttaaacttaagcttATGGAAAAGGGAACAGACAGAATG<br>R: tgctggatatctgcagaattcCTACACAGGCTTGCTACTAGTTTTGC	1803

Note: The lowercase letters in the table represent the added recombinant sequences.

Supplementary Table S2 Primer sequence used for qRT-PCR

Genes	Primer sequences (5'-3')
NCAPG	F: AAGAAGAGAGTGCTACAATTCACAG<br>R: CTTATCAGCCTCCCAGAAAACATC
LCORL	*F: TCCCCTAGTTGCTCAGGAATTAATG*<br>*R: ATTCTGTTCTTGAGTTCGATTCAC*
MyOG	F: CGGAGGCTGAAGAAGGTGAA<br>R: CGGTCCTCTGCCTGGTCAT
MyHC	F: CTCCTCACGCTTTGGTAA<br>R: TGATAGTCGTATGGGTTGGT
Myomaker	F: TGGGTGTCCCTGATGGC<br>R: CCCGATGGGTCCTGAGTAG
CDKN1A	F: CCCGTAGACCACGAGCAGAT<br>R: CGTCTCGGTCTCGAAGTTGA
CDKN2B	F: CACGGCTGCGGATGAACTAG<br>R: TCCGACCGAAGGAGTTGACAG
CCNB2	F: CCTCTTCCACTTCACTTCT<br>R: CTTTGTACCCCACTTATCA
GAPDH	F: GAACATCATCCCAGCGTCCA<br>R: CGGCAGGTCAGGTCAACAAC

Note: F means forward primer; R means reverse primer.

Supplementary Table S3. Chicken *NCAPG-LCORL* gene mutation site information and association results with BW8.

Chr	POS	SNP	REF	ALT	*P* (BW8)	Chr	POS	ID	REF	ALT	*P* (BW8)
4	75738569	rs75738569	C	T	0.0020	4	75813715	rs75813715	C	A	0.3694
4	75738570	rs75738570	G	A	0.0901	4	75813817	rs75813817	T	C	0.3694
4	75740715	rs75740715	T	A	0.1325	4	75813879	rs75813879	C	G	0.2426
4	75740734	rs75740734	C	T	0.5740	4	75817862	rs75817862	C	T	0.3650
4	75740760	rs75740760	T	C	0.0002	4	**75853958**	rs75853958	C	T	0.3516
4	75740767	rs75740767	G	T	0.0008	4	**75854181**	rs75854181	C	T	0.1610
4	75740777	rs75740777	A	G	0.9400	4	**75854199**	rs75854199	T	A	0.5882
4	75740781	rs75740781	T	C	0.9400	4	**75854226**	rs75854226	C	A	0.1610
4	75765375	rs75765375	C	T	0.0068	4	**75854244**	rs75854244	T	C	0.4854
4	75765412	rs75765412	A	G	0.0013	4	**75858968**	rs75858968	C	T	0.0081
4	75765550	rs75765550	T	C	0.0024	4	**75859000**	rs75859000	G	A	0.1262
4	75765554	rs75765554	T	A	0.0024	4	**75884042**	rs75884042	G	A	0.1725
4	75776836	rs75776836	T	C	0.0004	4	**75886144**	rs75886144	A	T	0.6225
4	75790556	rs75790556	T	C	0.0073	4	**75886188**	rs75886188	C	T	0.6225
4	75790557	rs75790557	A	G	0.0981	4	75913644	rs75913644	T	C	0.1893
4	75790569	rs75790569	A	G	0.0073	4	75913701	rs75913701	T	A	0.0138
4	75796968	rs75796968	T	C	0.1098	4	75913711	rs75913711	A	C	0.1927
4	75809014	rs75809014	C	T	0.7310	4	75914118	rs75914118	C	T	0.1443
4	75809041	rs75809041	A	G	0.1347	4	75915034	rs75915034	C	A	0.0048
4	75809070	rs75809070	G	A	0.1347	4	75915068	rs75915068	C	T	0.1793
4	75813512	rs75813512	T	C	0.1310	4	75915663	rs75915663	A	G	0.0568
4	75813669	rs75813669	T	C	0.1638	4	75915728	rs75915728	C	T	0.0061
4	75790569	rs75790569	A	G	0.0073	4	75913701	rs75913701	T	A	0.0138

Note: In bold are SNPs shared by the *NCAPG* and *LCORL* genes, the same below.
